# Successful Treatment of Refractory Asthma Clinically Diagnosed As Eosinophilic Granulomatosis With Polyangiitis in a Hairdresser Using Tezepelumab

**DOI:** 10.7759/cureus.68570

**Published:** 2024-09-03

**Authors:** Toyoshi Yanagihara, Fumiyasu Igata, Masaki Fujita

**Affiliations:** 1 Department of Respiratory Medicine, Fukuoka University Hospital, Fukuoka, JPN

**Keywords:** thymic stromal lymphopoietin (tslp), mepolizumab, tezepelumab, eosinophilic granulomatosis with polyangiitis (egpa), bronchial asthma

## Abstract

We report a case of a 53-year-old male hairdresser with refractory asthma clinically diagnosed as eosinophilic granulomatosis with polyangiitis (EGPA) who showed remarkable improvement with tezepelumab after failing mepolizumab therapy. The patient presented with a three-year history of progressive multisystem involvement, including anosmia, asthma, hearing loss, and skin rash. The patient was clinically diagnosed as EGPA based on asthma, sinusitis with nasal polyps, eosinophilia, and purpura. Despite initial improvement with oral corticosteroids and mepolizumab, he experienced recurrent exacerbations of asthma. Tezepelumab was initiated, resulting in significant symptom improvement, successful corticosteroid tapering, and marked enhancement in pulmonary function tests. This case suggests that tezepelumab may be an effective treatment option for patients with refractory asthma, particularly those with suspected occupational exposure. Further research is needed to identify factors that predict response to different biologic therapies in refractory EGPA-related asthma and to explore the potential role of occupational exposures in treatment outcomes.

## Introduction

Hairdressers face a significantly higher risk of developing occupational asthma due to prolonged exposure to various active chemicals [1-5]. These individuals commonly exhibit eosinophilic inflammation in their airways and develop dermatitis and rhinitis, suggesting immunologic mechanisms [3].

Eosinophilic granulomatosis with polyangiitis (EGPA) is a rare inflammatory disease affecting 0.5-4.2 individuals per million annually, and it is characterized by asthma, eosinophilia, and multi-organ involvement [6]. EGPA presents diagnostic and management challenges, often requiring a collaborative approach across medical disciplines.

Tezepelumab, a human monoclonal antibody, is a biologic treatment for severe asthma [7]. It works by blocking thymic stromal lymphopoietin (TSLP), an epithelial cytokine that plays a crucial role in initiating and sustaining airway inflammation in asthma [7]. By inhibiting TSLP, tezepelumab disrupts the protein's interaction with its receptor. This prevents the activation of downstream inflammatory cascades and mitigates effects on airway structural cells and hyperresponsiveness. This treatment has demonstrated efficacy in reducing the annualized asthma exacerbation rate, improving lung function and asthma control, and enhancing health-related quality of life in individuals with severe, uncontrolled asthma [8].

In this context, we present a case of a hairdresser with refractory asthma clinically diagnosed as EGPA who showed remarkable improvement with tezepelumab after failing mepolizumab therapy.

## Case presentation

A 53-year-old male hairdresser with no smoking history presented with a complex three-year medical history characterized by progressive multisystem involvement. The patient's initial symptom was anosmia, diagnosed as sinusitis by a local physician. This was followed by the development of cough and exertional dyspnea, leading to a diagnosis of bronchial asthma at another hospital and the initiation of budesonide/formoterol inhaler therapy. Two years prior to presentation, the patient developed hearing impairment, progressing to hearing loss within a year, necessitating bilateral myringotomy for presumed otitis media. Concurrently, his sinusitis was suspected to be eosinophilic in nature. Six months before admission, he developed a widespread rash, with a skin biopsy suggesting leukocytoclastic vasculitis.

The patient's respiratory symptoms intensified, prompting oral corticosteroid therapy, which temporarily improved his anosmia and respiratory symptoms. However, steroid discontinuation led to symptom exacerbation, including severe exertional dyspnea (mMRC grade 3), necessitating referral to our tertiary center for further evaluation and management. The patient's medical history was significant for a crab allergy. Medications at the initial presentation included fluticasone furoate/umeclidinium/vilanterol (Trelegy).

**Figure 1:**
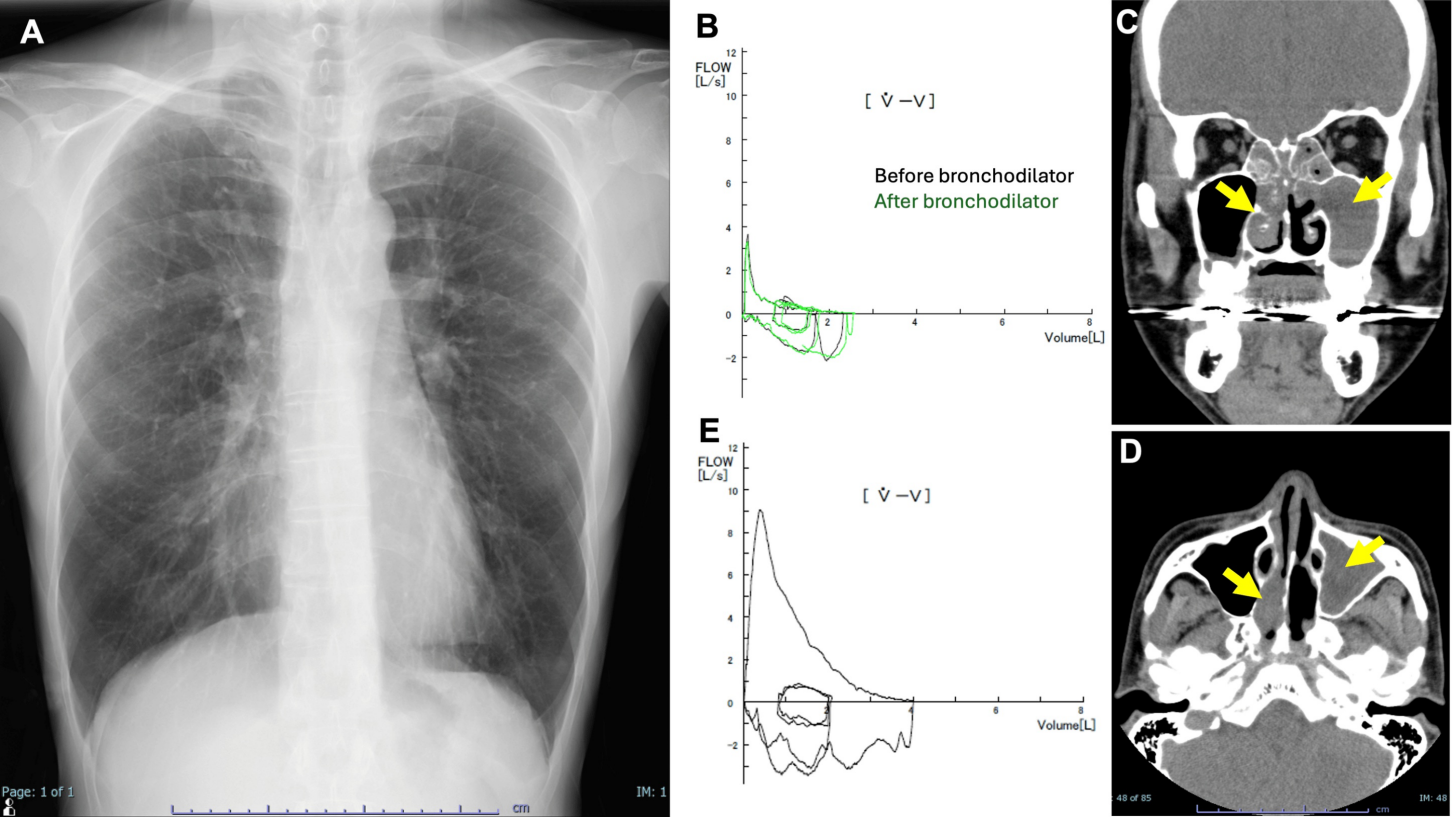
Radiographic and pulmonary function findings before and after treatment (A) Chest X-ray at initial presentation. (B) Flow-volume curve at initial presentation, demonstrating severe obstructive ventilatory impairment. The black line represents the flow-volume curve before bronchodilator administration, while the green line shows the curve after bronchodilator use. (C, D) CT of the paranasal sinuses demonstrated extensive soft tissue density areas filling the left maxillary sinus, extending to the ethmoid, sphenoid, and frontal sinuses (arrows). (E) Flow-volume curve eight months after initiation of tezepelumab treatment, indicating significant improvement in lung function. CT: computed tomography

On admission, vital signs were as follows: SpO2 90% (room air), respiratory rate 20/min, temperature 37.1°C, pulse rate 103/min, and blood pressure 132/94 mmHg. Auscultation revealed diffuse wheezing. Chest radiography demonstrated mild hyperinflation of the lungs, but no obvious parenchymal abnormalities were observed in the lung fields (Figure 1A). Urinalysis was negative for hematuria and proteinuria. Laboratory investigations showed the following: C-reactive protein 0.38 mg/dL, white blood cell count 8,600/µL with 7.8% eosinophils (absolute count 670/µL), and total IgE 689 IU/mL. Pulmonary function tests revealed severe obstructive ventilatory impairment: FVC 2.49L (59.4% predicted), FEV1 0.85L (23.9% predicted), and V25 0.11 L/s (5.7% predicted) (Figure 1B). Fractional exhaled nitric oxide was markedly elevated at 176 ppb.

Otolaryngological examination revealed bilateral nasal polyps, ethmoid sinus opacities, and pulsatile otorrhea from the right ear. CT of the paranasal sinuses demonstrated extensive soft tissue density areas filling the left maxillary sinus, extending to the ethmoid, sphenoid, and frontal sinuses (Figure 1C-1D). These findings suggest eosinophilic sinusitis and otitis media. The nasal polyp biopsy was deferred due to respiratory instability. The patient was diagnosed with refractory asthma complicated by suspected eosinophilic sinusitis and otitis media. EGPA was considered in the differential diagnosis. Myeloperoxidase anti-neutrophil cytoplasmic antibodies (MPO-ANCA) and rheumatoid factor were both negative.

Initial treatment with oral prednisolone (PSL) 30 mg showed limited improvement. Oxygen requirements increased to 2 L/min, necessitating a switch to intravenous betamethasone 16 mg. The patient's condition gradually improved, allowing for steroid tapering and discharge on a dose of PSL 30 mg.

**Figure 2:**
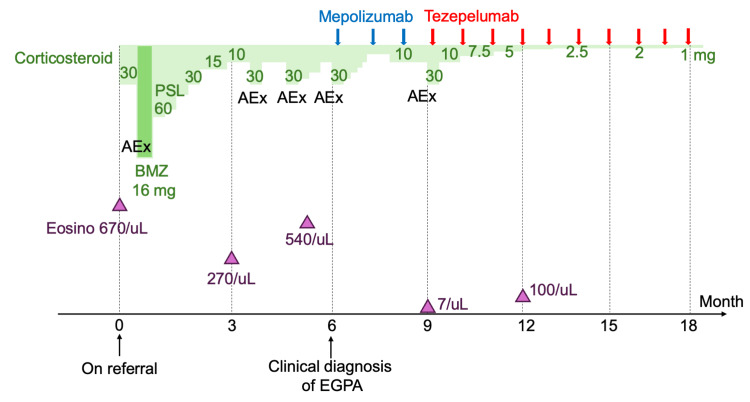
Clinical course of the patient with EGPA-related refractory asthma The graph illustrates the patient's treatment history and disease progression over 18 months. The green line represents corticosteroid dosage (mg/day) with initial high-dose PSL and BMZ treatment. Blue arrows indicate mepolizumab administration, while red arrows show tezepelumab treatment. Purple triangles represent eosinophil counts (/μL) at various time points. AEx are marked along the timeline. The x-axis shows months since referral. Corticosteroid doses were gradually tapered, with notable reduction following tezepelumab initiation. Eosinophil counts decreased significantly after biologic therapy initiation. EGPA: eosinophilic granulomatosis with polyangiitis, PSL: prednisolone, BMZ: betamethasone, AEx: acute exacerbations

Outpatient management involved further steroid tapering, but asthma exacerbations recurred when the dose was reduced to 10 mg, requiring repeated increases to 30 mg. Six months post-referral, the patient presented to the emergency department with an asthma exacerbation and skin purpura, leading to a clinical diagnosis of EGPA. Treatment was intensified with PSL 30 mg and subcutaneous mepolizumab 300 mg every four weeks, initially improving asthma control. However, nine months post-referral, respiratory symptoms worsened (SpO2 91%, wheezing) despite low peripheral eosinophil count (70/µL) and IgE (97 IU/mL). Mepolizumab was discontinued, and tezepelumab 210 mg every four weeks was initiated, resulting in significant symptom improvement. Ten months post-referral (one month after tezepelumab initiation), the patient was asymptomatic with SpO2 98%. Oral corticosteroids were successfully tapered without relapse. At 17 months post-referral (eight months after tezepelumab initiation), pulmonary function tests showed marked improvement: FVC 4.34L (104% predicted), FEV1 2.83L (80.9% predicted), and V25 0.41L/s (21.9%) (Figure 1E). At 19 months post-referral, the patient had regained his sense of smell and was free from asthmatic symptoms, skin manifestations, and otitis media while maintained on 1 mg of PSL. The patient's clinical course is described in Figure 2. The patient continued to work as a hairdresser. He took maximum precautions with ventilation and used an air purifier in his workspace to minimize exposure to potential triggers.

## Discussion

This case report demonstrates the successful use of tezepelumab in a hairdresser with refractory asthma clinically diagnosed as EGPA, which was unresponsive to mepolizumab treatment. While no direct association between hairdressing and EGPA development has been reported, hairdressers' frequent exposure to irritative and allergenic substances can lead to asthma [4,5]. Occupational asthma in hairdressers is primarily attributed to persulfate salts found in bleaching agents [3]. The underlying mechanism is complex and not fully elucidated, potentially involving both non-specific and specific immune responses [4]. Non-specific responses include direct irritation of airway mucosa and histamine release, while specific immune responses may involve IgE and non-IgE-mediated pathways. The latter is supported by observations of delayed-type reactions and eosinophilic inflammation in the airways. Interestingly, skin exposure to persulfates may play a role in respiratory sensitization, as evidenced by animal studies and the frequent occurrence of dermatitis preceding respiratory symptoms in hairdressers [4]. We speculate that tezepelumab's efficacy, in this case, might be attributed to its ability to block TSLP production resulting from epithelial irritation due to various chemical exposures in the hairdressing environment. By inhibiting TSLP, tezepelumab may effectively suppress the upstream signaling that leads to the recruitment and activation of various immune cells, including eosinophils.

The patient was clinically diagnosed with EGPA based on the Japanese criteria for EGPA [9], which includes pre-existing bronchial asthma, eosinophilia despite steroid use, and purpura as major clinical findings. The patient's clinical course, characterized by asthma and eosinophilia preceding purpura, further supported this diagnosis. The patient's condition was indeed certified under Japan's Designated Intractable Disease System, a national program that provides medical and financial support for patients with rare and severe chronic diseases. Furthermore, the patient met the 2022 American College of Rheumatology/European Alliance of Associations for Rheumatology (ACR/EULAR) Classification Criteria for EGPA [10], scoring 6 points (asthma +3, nasal polyps +3). However, it's important to note that we did not have histological evidence of eosinophilic infiltration or granulomatous or fibrinoid necrotic vasculitis of small vessels in the patient. Atypically, the patient did not exhibit neurological symptoms, which are present in over 90% of Japanese EGPA cases [11]. This necessitates careful monitoring of the patient's clinical course.

Management of severe EGPA-related asthma remains challenging, with up to 50% of patients experiencing persistent airflow obstruction [12,13]. While monoclonal antibodies such as mepolizumab (targeting interleukin-5) and benralizumab (targeting the interleukin-5 receptor α subunit) have demonstrated efficacy in clinical trials for EGPA [14,15], their effectiveness remains limited. The MIRRA study showed that over 50% of patients receiving mepolizumab did not achieve the primary outcome [14]. Real-world retrospective studies in France have reported similar findings for mepolizumab and benralizumab in patients with severe EGPA-related asthma [16]. These studies used a key outcome measure of patients achieving a daily oral prednisone dose of 4 mg or less, coupled with a Birmingham Vasculitis Activity Score (BVAS) of 0 at 12 months. Only 32% of patients met this primary outcome after a year of treatment, with 14% experiencing asthma exacerbations during treatment, highlighting the persistent challenges in managing this condition.

TSLP is recognized as a crucial upstream driver of type 2 inflammation, and genetic variants leading to increased TSLP protein secretion have been associated with a higher risk of EGPA [17]. Despite this potential link, there is only one published case series demonstrating tezepelumab treatment for EGPA-related asthma [18]. This series described two ANCA-negative patients who had been treated with mepolizumab and benralizumab but experienced decreased efficacy before switching to tezepelumab. One case showed marked improvement, while the other showed a partial response.

Limitations of this case report include the lack of quantitative measures such as BVAS or ACT scores. Additionally, due to the COVID-19 pandemic, we were unable to perform frequent pulmonary function tests, including measurements before and after mepolizumab treatment.

## Conclusions

This case suggests that tezepelumab may be an effective treatment option for patients with refractory asthma, particularly those with suspected occupational exposure-related symptoms. While the patient was clinically diagnosed with EGPA, the atypical presentation warrants careful follow-up. Further research is needed to identify factors that predict response to different biologic therapies in refractory EGPA-related asthma and to explore the potential role of occupational exposures in treatment outcomes.
